# Health-related quality of life, functional decline, and long-term mortality in older patients following hospitalisation due to COVID-19

**DOI:** 10.1186/s12877-021-02140-x

**Published:** 2021-03-22

**Authors:** M. M. Walle-Hansen, A. H. Ranhoff, M. Mellingsæter, M. S. Wang-Hansen, M. Myrstad

**Affiliations:** 1 Department of Medical Research, Bærum Hospital, Vestre Viken Hospital Trust, N-1346 Gjettum, Norway; 2 Department of Medicine, Diakonhjemmet Hospital, Oslo, Norway; 3 Department of Clinical Science, University of Bergen, Bergen, Norway; 4 Department of Geriatric Medicine, Akershus University Hospital, Lørenskog, Norway; 5 Department of Geriatric Medicine, Vestfold Hospital Trust, Tønsberg, Norway; 6 Department of Internal Medicine, Bærum Hospital, Vestre Viken Hospital Trust, N-1346 Gjettum, Norway

**Keywords:** COVID-19, Health-related quality of life, Geriatric medicine, Pandemic

## Abstract

**Background:**

Older people are particularly vulnerable to severe COVID-19. Little is known about long-term consequences of COVID-19 on health-related quality of life (HR-QoL) and functional status in older people, and the impact of age in this context. We aimed to study age-related change in health-related quality of life, functional decline and mortality among older patients 6 months following hospitalisation due to COVID-19.

**Methods:**

This was a cohort study including patients aged 60 years and older admitted to four general hospitals in South-Eastern Norway due to COVID-19, from March 1 up until July 1, 2020. Patients who were still alive were invited to attend a six-month follow-up. Change in HR-QoL and functional status compared to before the COVID-19 hospitalisation were assessed using the EuroQol 5-dimensional-5 levels questionnaire (EQ. 5D-5L). A change in visual analogue scale (VAS) score of 7 or more was considered clinically relevant.

**Results:**

Out of 216 patients aged 60 years and older that were admitted to hospital due to COVID-19 during the study period, 171 were still alive 180 days after hospital admission, and 106 patients (62%) attended the six-month follow-up. Mean age was 74.3 years, 27 patients (26%) had experienced severe COVID-19. Fifty-seven participants (54%) reported a decrease in the EQ. 5D-5L VAS score after 6 months, with no significant difference between persons aged 75 years and older compared to younger. Seventy participants (66%) reported a negative change in any of the dimensions of the EQ. 5D-5L, with impaired ability to perform activities of daily life (35%), reduced mobility (33%) and having more pain or discomfort (33%) being the most commonly reported changes. Forty-six participants (43%) reported a negative change in cognitive function compared to before the COVID-19 hospitalisation. Six-month mortality was 21%, and increased with increasing age.

**Conclusions:**

More than half of the patients reported a negative change in HR-QoL 6 months following hospitalisation due to COVID-19, and one out of three experienced a persistently impaired mobility and ability to carry out activities of daily living. The results suggest awareness of long-term functional decline in older COVID-19 patients.

**Supplementary Information:**

The online version contains supplementary material available at 10.1186/s12877-021-02140-x.

## Background

The acute respiratory syndrome coronavirus 2 (SARS-CoV-2) has caused a pandemic with over 83 million confirmed cases and over 1.8 million deaths globally by January 3, 2021 [1].

The number of deaths and confirmed cases of coronavirus disease (COVID-19) differ dramatically between countries. Norway, with its 5.5 million inhabitants has had a relatively low number of confirmed cases. By December 1, 2020 the cumulative number of people admitted to hospital with COVID-19 in Norway was 1728 [2]. In concordance with international data, the majority of hospitalised patients have been aged 60 years and older [3].

Old age and comorbidities increase the risk of severe COVID-19 and constitute the most prominent risk factors for in-hospital mortality due to COVID-19 [4, 5].

COVID-19 primarily affects the lungs and the majority of hospital patients present with respiratory symptoms and fever [6]. In older people, acute functional decline, confusion and asthenia are also commonly reported symptoms [7, 8]. COVID-19 causes hypoxemia, and prolonged oxygen treatment requirement is associated with increased length of hospitalisation, with risk of immobilisation and acute sarcopenia [9]. In general, immobilisation during acute illness can cause loss of physical functions with impact on activities of daily living (ADL) [10, 11]. In some older people, hospitalisation causes persistent functional decline [12]. So far, few studies have reported long-term consequences of COVID-19 [13]. The role of age for functional decline in older people who survive acute and severe disease is largely unknown. Increased knowledge about determinants of functional capacity following hospitalisation due to COVID-19, is imperative to develop preventive measures in patients at risk.

Cognitive and physical function are among the most important factors for quality of life and independent living in older people. Health-related quality of life (HR-QoL) is an important measure used to assess patients’ perception of the impact of disease and disability on different dimensions of health [14]. A few studies suggest the pandemic might influence HR-QoL in general, and that older people might have higher risk of experiencing reduced HR-QoL during the pandemic than younger [15]. To our best knowledge, only a few studies have reported post-discharge symptoms and HR-QoL in patients hospitalised due to COVID-19 [13, 16, 17]. Long-term changes in HR-QoL, functional status and the ability to carry out ADL in older people following hospitalisation due to COVID-19, have been sparsely investigated until now.

In a six-month follow-up, we aimed to study age-related change in HR-QoL, functional status and mortality among patients aged 60 years and older after hospitalisation due to COVID-19.

## Methods

### Study population

This was a multi-centre cohort study including patients aged 60 years and older who were admitted to four general hospitals in South-Eastern Norway due to COVID-19 from March 1 up until July 1, 2020. The patients were identified from administrative hospital data. All patients who were still alive 180 days after hospital admission were invited to participate in the study.

The participating centres were Akershus University Hospital, Bærum Hospital Vestre Viken Hospital Trust, Diakonhjemmet Hospital and Vestfold Hospital Trust. Together, the four hospitals serve about 1,125,000 inhabitants in the Oslo Region, Norway. This comprises around 20% of the total Norwegian population.

COVID-19 was diagnosed by qualitative detection of nucleic acid from SARS-CoV-2 in throat or nasal secretions by use of real-time polymerase chain reaction. Patients with SARS-CoV-2 without symptoms of COVID-19 hospitalised for any other reasons, were not considered for inclusion. All eligible patients received an invitation letter to participate in the study and to attend a follow-up consultation at their respective hospital around 6 months after hospitalisation. For disabled patients, home visits or visits to nursing homes were arranged.

### Measurements

Characteristics of the participants, including comorbidities and work status were obtained from hospital records. We registered the length of hospitalisation, and whether the patient had experienced severe or critical acute COVID-19 illness, defined as admission to an intensive care or intermediary care unit (ICU).

HR-QoL and functional status 6 months after hospitalisation were assessed using the EuroQol 5-dimensional-5 levels (EQ. 5D-5L) questionnaire [18]. The EQ. 5D-5L includes a vertical visual analogue scale (VAS) from 0 to 100 rating overall HR-QoL. Furthermore, the tool assesses functional status with five dimensions (*mobility, self-care, usual activities, pain/discomfort and depression/anxiety*), each with five levels of function (*no problems, slight problems, moderate problems, severe problems and unable to/extreme problems*).

Premorbid HR-QoL and functional status (defined as 2 weeks prior to COVID-19 symptom onset) were reported retrospectively at 6 months after hospital admission. The majority of patients replied to the premorbid HR-QoL at home before attending the follow-up consultation, while status after 6 months was filled out as a part of the study visit. For disabled and cognitively impaired patients, the HR-QoL questionnaires were answered with the help of a next-of-kin.

Patient-reported outcome measure on change in cognitive function compared to 2 weeks before the onset of COVID-19 symptoms, was assessed with the following question: *Has your cognitive function changed?* (Additional file 1 p 3).

We used the Montreal Cognitive Assessment (MoCA) to evaluate cognitive capacity, and the Short Physical Performance Battery (SPPB) to assess functional capacity [19, 20]. Height and weight were measured as a part of the study visit.

In-hospital mortality was defined as death during the index hospital stay, related or unrelated to COVID-19, and was obtained from hospital records. Six-month mortality was defined as death related or unrelated to COVID-19 within 180 days after the index hospital admission date. Mortality status 180 days after hospital admission was based on reports from hospital electronic patient record systems, which update weekly from the Norwegian National Death Register.

### Statistical methods

We used the registry tool EpiData entry client version 4.4.3.1 (The EpiData Association, Odense, Denmark). Continuous variables are presented as mean ± standard deviation and categorical variables as number (%). We used Student’s t test for means of continuous variables and Pearson’s Chi-square test of independence for categorical variables to compare characteristics of study participants aged under 75 years with participants aged 75 years and older. *P*-values < 0.05 were considered statistically significant.

We aimed to compare long-term outcomes between the oldest patients and the younger old patients in the study. Age is an independent risk factor for poor outcome in patients hospitalised due to COVID-19 [21], and a cut-off of 75 years was chosen based on clinical experience and with the aim to create subgroups of equal size.

We present change in HR-QoL as the mean change in EQ. 5D-5L VAS (0–100) by age group and disease severity, as well as the percentage of survivors reporting a minimally clinically important difference in scores before compared to after hospitalisation due to COVID-19. We defined a clinically important difference as a change in the VAS of 7 or more [22]. We present change in functional status as the mean change in each of the five EQ. 5D-5L dimensions (mobility, self-care, usual activities, pain and anxiety) by age group, as well as the percentage of survivors reporting worsened function for each of the dimensions.

In-hospital mortality and six-month mortality was calculated with the total number of patients over 60 years of age who were hospitalised due to COVID-19 during the study period as denominator, and is reported as % by age group. We present six-month mortality by age group as Kaplan-Meier plots.

All statistical analyses were conducted using SPSS version 26.0 (IBM, Armonk, NY, USA).

### Ethical considerations

Patients who were alive at six months after hospitalisation, and able to receive and understand information about the study, signed a written informed consent to participate. If the patient was not able to consent, a passive consent to participate was assumed. The patient’s proxy was then informed about the study and could withdraw participation on behalf of the patient. The need for written informed consent from the proxy of patients who were unable to consent was waived by the Norwegian Regional Committees for Medical and Health Research Ethics (REC Central, reference number 155425), due to the retrospective nature of the study, and that participation would imply minimal risk or disadvantage for the participants. Patients who died were included with patient administrative data only, and the need for informed consent was waived by the REC Central (reference number 155425) due to the retrospective nature of the study. Participants or next-of-kin were at any time able to withdraw from the study.

The study was approved by the Norwegian Regional Committees for Medical and Health Research Ethics (REC Central, reference number 155425) and the officers of data protection at all the participating centres, and complies with the Declaration of Helsinki.

## Results

**Fig. 1:**
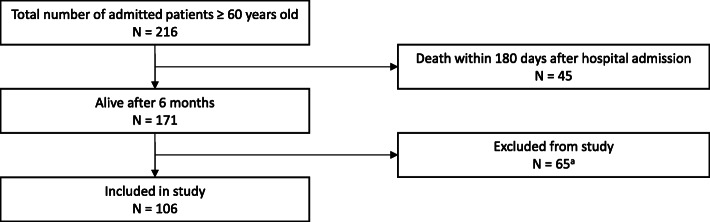
Inclusion of participants to the study. ^a^ 19 patients did not wish to participate in the study, 8 patients we were unable to reach by phone, 1 patient died before the follow-up evaluation and 2 patients were excluded because of severe cognitive and physical impairment, after consensus between the first and last author. Due to capacity challenges during the first wave, the list of admitted patients was retrospectively found to be incomplete for one of the participating hospitals, and 35 patients were therefore not invited to participate in the study

In total, 216 patients aged 60 years and older were hospitalised due to COVID-19 during the study period. Out of 171 persons who were still alive 6 months after hospital admission and eligible for the study, 106 (62%) attended the six-month follow-up (median 186 days after discharge). Figure 1 shows the inclusion of participants to the study.

### Study population

Table 1 shows characteristics of the study population by age group. Mean age was 74.3 (range 60–96) years and 60 patients (57%) were men. Twenty-seven patients (26%) had experienced severe or critical acute COVID-19, defined as ICU or intermediary unit care during hospitalisation. The mean length of hospitalisation was 11.6 (range 1–67) days. The proportion with severe COVID-19 was higher in the youngest age group. Comorbid conditions were more prevalent in the oldest age group. The mean sum scores of both MoCA and SPPB were lower in the oldest age group, indicating lower cognitive and physical function in older compared to younger participants.

**Table 1:** Study population characteristics at six-month follow-up by age group

	< 75 years old ***n*** = 61	≥ 75 years old ***n*** = 45	***p***-value
Age (mean, SD)	68.4 (4.6)	82.4 (5.3)	< 0.05
	n (%)	n (%)	
Male gender	37 (61)	23 (51)	0.33
Current or previous smoking^a^	30 (50)	26 (59)	0.36
Severe acute disease^b^	22 (36)	5 (11)	< 0.05
Length of hospital stay (mean, SD)	12.5 (12.5)	10.3 (12.1)	0.37
**Work status**^c^			< 0.05
Full-time or part-time employed	18 (30)	0 (0)	
Sick leave, retirement or disability advantages	42 (69)	45 (100)	
**Comorbidities**			
Hypertension	22 (36)	19 (42)	0.52
Cardiac conditions	12 (20)	22 (49)	< 0.05
COPD^d^ or asthma	18 (30)	11 (24)	0.56
Diabetes mellitus	10 (16)	6 (13)	0.66
Dementia	0 (0)	5 (11)	< 0.05
Obesity (BMI > 30)^e^	16 (28)	6 (16)	0.20
Other^f^	9 (15)	14 (31)	< 0.05
**Cognitive capacity**	Mean (SD)	Mean (SD)	
MoCA total score (0–30)^g^	25.3 (3.8)	21.7 (5.8)	< 0.05
**Functional capacity**			
SPPB total score (0–12)^h^	10.6 (2.3)	7.8 (3.1)	< 0.05

Patients aged 60 years and older admitted to four Norwegian hospitals from March 1 to July 1, 2020 due to COVID-19, and still alive after 6 months, *n* = 106

^a^Missing data for 2 patients

^b^Admission to intensive care unit or intermediary ward during hospitalisation

^c^Missing data for 1 patient

^d^Chronic obstructive pulmonary disease

^e^Missing data for 11 patients

^f^Including active cancer, previous depression, chronic kidney disease or previous brain stroke

^g^Incomplete or missing data for 14 participants

^h^Six patients did not perform the SPPB

### HR-QoL, functional decline and patient-reported outcome measures

**Fig. 2:**
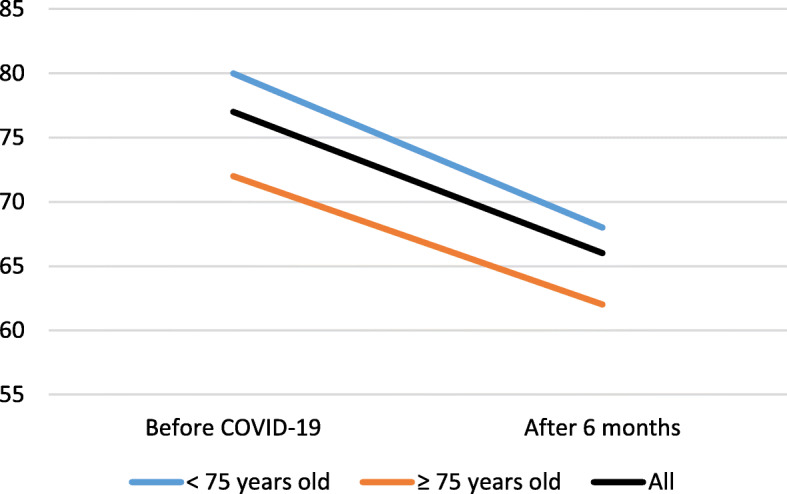
Mean change in HR-QoL (0–100 scale) by age 6 months after COVID-19. Patients aged 60 years and older admitted to four Norwegian hospitals from March 1 to July 1, 2020 due to COVID-19, and still alive after 6 months, *n* = 106. For 6 participants, information on EQ. 5D VAS scale was incomplete: 4 participants missed both status before and 6 months after COVID-19, and 2 participants missed status after 6 months

Out of 106 participants, 57 (54%) reported a decrease of more than 7 points in the EQ. 5D-5L VAS score, compared to before the hospital admission. Mean VAS score was 77.0 (SD 16.7) before admission and 65.8 (SD 19.1) after 6 months (mean change -11.5 (SD 14.2)). Figure 2 shows mean changes in the EQ. 5D-5L VAS by age group. Older people had lower scores both before and after COVID-19, but the magnitude of change did not differ between persons aged 75 years and older and younger people (mean change -10.3 (SD 12.8) vs. -12.3 (SD 15.1), *p* = 0.51). The share of patients who experienced a minimally clinically important change in VAS score did not differ between patients with severe or critical acute COVID-19 and less severe COVID-19 (68 vs. 53%, *p* = 0.20), but the magnitude of change was significantly larger among patients with severe or critical disease (mean change -17.6 (SD 16.2) vs. -9.5 (SD 13.0), *p* < 0.05).

While 35 (33%) and 37 patients (35%), respectively, reported a decline in mobility and the ability to perform activities of daily living compared to before COVID-19, 35 patients (33%) reported having more pain or discomfort, 28 patients (26%) increased anxiety and 18 patients (17%) a decline in the ability in self-care. Eleven patients (10%) reported a major change (a decrease of two or more functional levels) in mobility and 12 patients (11%) in usual activities. Table 2 demonstrates mean change in EQ. 5D-5L dimensions by age group.

**Table 2:** Mean change in EQ. 5D-5L dimensions by age group

	< 75 years old ***n*** = 61	≥ 75 years old ***n*** = 45	***p***-value
**Dimensions**^**a**^	Mean (SD)	Mean (SD)	
**Mobility**			
Mean score before COVID-19	1.3 (0.7)	1.6 (0.9)	0.15
Mean score after 6 months	1.8 (1.0)	2.0 (1.0)	0.32
Mean change	−0.4 (0.8)	−0.4 (0.8)	0.87
**Self-care**			
Mean score before COVID-19	1.1 (0.4)	1.4 (0.8)	< 0.05
Mean score after 6 months	1.3 (0.7)	1.6 (1.0)	0.06
Mean change	−0.2 (0.6)	−0.3 (0.7)	0.68
**Usual activities**			
Mean score before COVID-19	1.3 (0.6)	1.7 (1.1)	< 0.05
Mean score after 6 months	1.7 (1.1)	2.2 (1.2)	< 0.05
Mean change	−0.5 (0.9)	− 0.5 (0.9)	0.68
**Pain**			
Mean score before COVID-19	1.7 (0.8)	1.8 (0.9)	0.36
Mean score after 6 months	2.0 (0.9)	2.1 (0.9)	0.40
Mean change	- 0.3 (0.6)	−0.3 (0.6)	0.96
**Anxiety**			
Mean score before COVID-19	1.2 (0.5)	1.4 (0.8)	0.06
Mean score after 6 months	1.5 (0.8)	1.8 (1.1)	0.16
Mean change	−0.4 (0.6)	−0.3 (0.8)	0.70

Patients aged 60 years and older admitted to four Norwegian hospitals from March 1 to July 1, 2020 due to COVID-19, and still alive after 6 months, *n* = 106

^a^Incomplete data for 3 participants: 1 participant missing premorbid level of self-care, 1 participant missing premorbid and 6 months status for pain, as well as premorbid score for anxiety, and finally 1 participant missing premorbid and 6 month status for anxiety

**Fig. 3:**
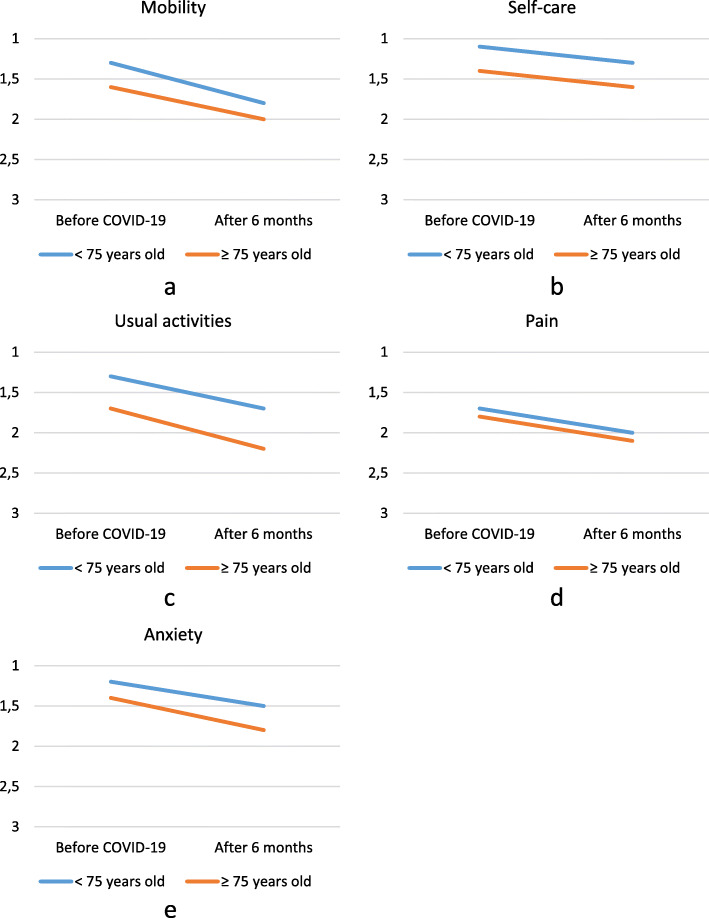
**a-e** Change in EQ. 5D-5L dimensions after hospitalisation due to COVID-19 by age group. Patients aged 60 years and older admitted to four Norwegian hospitals from March 1 to July 1, 2020 due to COVID-19, and still alive after 6 months, *n* = 106

Figures 3a-e demonstrate changes in dimension-specific EQ. 5D-5L scores by age group. Older people had lower functional status, but the change in functional status did not differ between the age groups in any of the dimensions.

Forty-six of the participants (43%) experienced a negative change in cognitive function 6 months after the COVID-19 hospitalisation, with a a higher proporton reporting cognitive decline among persons 75 years and older, compared to younger persons (59% vs. 37%, *p* < 0.05).

### Mortality

**Fig. 4:**
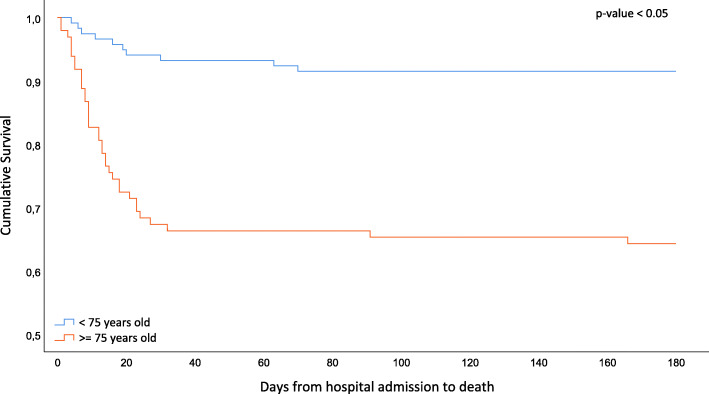
One hundred and eighty day survival after hospitalisation due to COVID-19 by age group. Patients aged 60 years and older admitted to four Norwegian hospitals from March 1 to July 1, 2020, due to COVID-19, *n* = 216

In-hospital mortality was 16% (34 out of 216 patients). Six-month mortality was 21% (45 out of 216 patients), 36% in patients aged 75 years and older (35 out of 98 patients) and 8% in patients aged 60 to 74 years (10 out of 118 patients) (*p* < 0.05). Eleven patients (5%) died out of hospital. Figure 4 shows Kaplan-Meier survival plots by age group.

## Discussion

To our knowledge, this is the first study to report long-term impact of COVID-19 on HR-QoL and functional status in older people.

More than half of the study participants reported a clinically relevant decline in HR-QoL 6 months after hospitalisation due to COVID-19, compared to before hospital admission. One out of three patients reported decline in mobility and the ability to carry out activities of daily living. We found no difference in functional decline 6 months after hospitalisation due to COVID-19 when comparing patients aged 75 years or older with patients aged under 75 years. Still, the oldest patients reported lower functional status, possibly increasing their risk of permanent care needs and loss of independence following hospitalisation due to COVID-19. The results of our study suggest awareness of long-term functional decline in older COVID-19 patients, and sends the message to prevent both the disease and its complications in this vulnerable group of patients.

Reports about persistent symptoms among patients who have suffered from COVID-19 are emerging, and studies of long-term consequences of the disease are highly warranted [23]. So far, knowledge on HR-QoL in survivors is very sparse [17], and we are not aware of any studies that have studied older patients with this regard.

In a recently published paper, Huang, et al., reported persistent symptoms among patients with a median age of 57 years, after hospitalisation due to COVID-19 in China [13]. Interestingly, the mean EQ. 5D-5L VAS at 6 months was much lower in our study compared to in the patients in China (65.8 vs. 80.9), and very few patients in the Chinese study had problems with mobility or ADL. The most likely explanations for these differences are the much higher mean age, more comorbidity, and a higher proportion of patients with severe disease and ICU treatment, in our study. Patients who had experienced severe COVID-19 had the largest decline in HR-QoL in our study. The results suggest that special attention regarding long-term reduction of HR-QoL and functional decline should be drawn to the oldest patients and patients with severe COVID-19.

Functional loss during hospitalisation due to acute illness is common in older people. It reduces their ability to live independently, and increases the risk of nursing home admission and needs of community care [24–26]. In a recently published study that included 346 patients aged 65 years and older with confirmed seasonal influenza and other acute respiratory illness, 18% experienced persistent functional decline 30 days after discharge from hospital [12]. The functional losses were equivalent between patients with influenza and other acute respiratory illnesses.

Comparison of functional decline following acute illness between studied populations is complicated by variations in the used assessment methods and duration of follow-up. In a recently published meta-analysis, the pooled prevalence of hospital-associated disability among older adults was 30% [27]. However, all studies included in this analysis had a shorter follow-up period compared to our study. Whether COVID-19 might cause more severe long-term reduction in HR-QoL and functional capacity compared to other acute conditions, remains an unanswered question that should be addressed in further studies.

Many patients with COVID-19 require long-term oxygen treatment, and both persistent hypoxemia and isolation measures might promote immobilisation and delirium. Immobilisation due to acute illness increases the risk of acute sarcopenia and loss of physical function [9]. Sarcopenia is furthermore associated with negative impact on HR-QoL [28]. Emerging evidence suggest that older patients with COVID-19 are at high risk of developing delirium, a condition associated with both increased mortality and long-term cognitive impairment and dementia [29, 30].

Prevention of hospital admission due to COVID-19 with vaccination of older people might be an effective measure to prevent long-term functional decline. Furthermore, management of complications such as immobilisation and delirium, might prevent long-term functional loss, and should be implemented subsequently in older patients hospitalised with COVID-19.

### Strengths and limitations

The main strengths of this study is the long follow-up generating new knowledge about the persisting impact of the lives of older people who have survived severe COVID-19. Furthermore, the study population represents a high proportion of older survivors after hospitalisation due to COVID-19 in Norway. Also, the multicentre design supports the generalisability of our results.

This study has some limitations. We made use of the EQ. 5D-5L response form, which was scored retrospectively for the premorbid status at 6 months. The response in premorbid status might be influenced by experiences during hospitalisation, the patient’s current health status or the psychosocial effect of the pandemic situation in itself, leading to under- or overreporting of symptoms. A few studies have investigated the validity of retrospectively collected EQ. 5D-scores in other patient groups. These studies find no significant differences in retrospectively and prospectively reported EQ. 5D-scores on a population level [31, 32]. To prevent potential bias from patients reporting only small changes in the EQ. 5D VAS scale, we chose a cut-off value of 7 points as the minimally clinically important difference when comparing scores before and after discharge from hospital [22].

Comparison of characteristics across chronological age does not take into account the complex interplay of physiological, pathological and psychosocial changes seen with biological aging. Out of practical reasons, we chose to compare patients across chronological age in our study. Additional clinical characteristics of our study population might have elaborated this topic.

Patients were evaluated at a single point follow-up at 6 months. We experienced that some patients with a high degree of comorbidity or anamnestic great loss of function after COVID-19 were unwilling to participate in the study because of the resources demanded in meeting at the hospital. Home visits or institutional consultations were arranged to include as many of these patients as possible, but we still suspect that the frailest group is underrepresented in our study, potentially leading to underestimation of both symptoms and the magnitude of functional decline at 6 months. Comorbidities were most prevalent among the youngest age group in our study. This might be due to selection effect of survivors.

## Conclusions

This study is among the first to address long-term change in HR-QoL and functional decline among older people hospitalised due to COVID-19. More than half of patients aged 60 years and older reported a negative change in HR-QoL 6 months after hospitalisation due to COVID-19. One out of three experienced a persistent loss of physical function and ability to carry out ADL, with no difference between the age groups studied. Six-month mortality increased with increasing age, and was very high among patients 75 years or older. These results highlight the importance of preventing COVID-19 in older people, and to prevent functional decline in older patients hospitalised due to COVID-19.

## Supplementary Information

**Additional file 1:.** Symptom Questionnaire.

## Data Availability

The datasets used and/or analysed during the current study are available from the corresponding author on reasonable request.

## Abbreviations

ADL: Activities of daily living
COVID-19: Coronavirus disease
EQ. 5D-5L: EuroQol 5-dimensional-5 levels questionnaire
HR-QoL: Health-related quality of life
ICU: Intensive Care Unit
MoCA: Montreal Cognitive Assessment
SARS-CoV-2: Acute respiratory syndrome coronavirus 2
SPPB: Short Physical Performance Battery
VAS: Visual Analogue Scale
