# Supplementary material for: Health-related quality of life, functional decline, and long-term mortality in older patients following hospitalisation due to COVID-19
Source: BMC Geriatr. 2021 Mar 22;21:199. doi: 10.1186/s12877-021-02140-x (PMC7983098; doi:10.1186/s12877-021-02140-x)
Supplement: Supplementary file 1 — Additional file 1:. Symptom Questionnaire. [file 12877_2021_2140_MOESM1_ESM.docx]

**Additional file 1**

**Health-related quality of life, functional decline, and long-term mortality in older patients following hospitalisation due to COVID-19**

Walle-Hansen MM^1^, Ranhoff AH^2, 3^, Mellingsæter M^4^, Wang-Hansen MS^5^, Myrstad M^1, 6^

**^1^** Department of Medical Research, Bærum Hospital, Vestre Viken Hospital Trust, N-1346 Gjettum, Norway. Corresponding author: mail: marte@hansencorp.eu

^2^ Department of Medicine, Diakonhjemmet Hospital, Oslo, Norway

^3^ Department of Clinical Science, University of Bergen, Bergen, Norway

^4^ Department of Geriatric Medicine, Akershus University Hospital, Lørenskog, Norway

^5^ Department of Geriatric Medicine, Vestfold Hospital Trust, Tønsberg, Norway

^6^ Department of Internal Medicine, Bærum Hospital Vestre Viken Hospital Trust, N-1346 Gjettum, Norway

**Table of contents**

Symptom Questionnaire …………………………………………………………………………………………………….. 2

**Symptom Questionnaire**

1. Has your cognitive function changed?

- Yes, I have experienced a change in cognition
- No, I have not experienced a change in cognition
